# Epistemic responsibility: toward a community standard for human-AI collaborations

**DOI:** 10.3389/frai.2025.1635691

**Published:** 2025-07-04

**Authors:** Dan Lloyd

**Affiliations:** Department of Philosophy, Trinity College, Hartford, CT, United States

**Keywords:** artificial intelligence (AI), epistemology, research standards, research ethics, publication standards

P(doom), the probability of Artificial Intelligence destroying civilization, has recently emerged as a topic of public debate (https://en.wikipedia.org/wiki/P(doom); Thomas, 2024). The alleged probable causes of an AI apocalypse are variable, often inflected with science fiction tropes, but express and reveal underlying anxieties about the intrusion of AI into domains formerly reserved for humans (Kokotajlo et al., 2025). AI anxieties generally radiate from two sources: ethics, and epistemics. The creators of AI struggle to keep their systems “aligned” with human interests and values, ethical concerns (Christian, 2020). They also struggle to control AI “hallucinations”—which might be better described as confabulations, an epistemic challenge (Sun et al., 2024). If LLMs were compulsive liars, they could be rejected as sources of knowledge, regardless of their trappings of sincerity and conviction. But in several recent cases, AI systems have genuinely contributed to human knowledge, leveraging fast processing of big data and neural network pattern detection. This exploding area of AI application includes biomedical research, especially drug discovery (Gao et al., 2024; Jiang and Zhao, 2025), astronomy and cosmology (Huang et al., 2024; Spindler, 2022), and data analysis in social science (Balla et al., 2025). The newer releases of “agentic” and “reasoning” systems have afforded researchers (and, in effect, everyone) possibilities for usefully harnessing the particular expertise of AI for many applications (Mollick, 2025). This has exacerbated worries of cheating, by presenting AI productions as one's own original work, along with the threat of AI-created misinformation. These worries lurk at every level of education, and among academics, artists, scientists, and other professions.

While cheating and unwarranted credulousness are irresponsible, a simple proscription against the use of AI in education and the professions would preemptively block potentially beneficial applications for AI. In any case, it's increasingly evident that such a blanket prohibition will not work (Mello, 2023). What is needed instead is a positive code defining the proper use of AI in contexts where truth is to be discovered, preserved, extended, and communicated. These “knowledge contexts” are the domain of epistemic responsibility. The goal of this short opinion essay is to outline some principles that might be applied by researchers and serve as a standard for evaluation of good practice in academic and professional writing.

“Epistemic responsibility” is a broad topic, linking to philosophical discussions of the ethics of belief-formation and to the intellectual virtues supportive of the production of knowledge (Battaly, 2008; Meylan, 2013). The concern in the present essay is narrower. Here, the question is not whether AI should be recruited or avoided in the pursuit of knowledge. AI, we've seen, is already broadly in play across every intellectual domain and in most workplaces. Nor do these guidelines suggest how AI should be used, nor how AI-produced claims should be understood and evaluated. Rather, these guidelines aim to secure a precondition for these larger issues. Here, the question is how to acknowledge and make explicit AI-generated content. The guidelines below aim to demarcate the human and the artificial in publication; once the two are clearly distinguished, the larger questions of evidence and warrant may also find new illumination. This, however, is a topic for a future discussion.

The goals of the proposed protocols for AI collaborations are *transparency* and *replicability*. The two goals reinforce each other. Where one is achieved, the other usually is facilitated as well. These are standard goals in academic publication and scientific research. Providing a conspicuous standard protocol for AI use will help reassure readers and consumers about the good practices of authors, and will provide authors with a clear and visible standard of conduct, requiring full disclosure of any AI generated material in published research. These standards do not need to be ratified by professional organizations or publishers, although they could be. Rather, they will hopefully spread to become a simple community expectation. Their widespread use will enable AI to be used effectively but with maximum transparency.

The proposed standards are listed below, with a notional example (Box 1).

Standard 1: *prominence*: the inclusion of AI content must be immediately apparent to all readers, even at a first glance. The AI source needs to be stated in title header text, in as much detail as possible. As displayed in the notional example (Box 1), this statement does not imply AI co-authorship, but is a separate line item. This includes identifying sections of the paper drawing on AI output, and demarcation of text composed by AI. The paper abstract needs to include this information as well. If there is no AI content, this too should be stated.
Standard 2: *replicability*: AI in research applications inevitably involves shaping the behavior of the AI in service of the researcher. This is “prompt engineering,” and the prompt used is an essential tool for understanding the result, its implications, and its limitations. The operative prompts should be explicitly and fully stated in any work including AI generated content. The actual prompt(s) behind the AI content is also essential for replication. Ideally, stages of prompt evolution should be documented, and submitted as Supplementary material (if not stated fully in the main text).
Standard 3: *content cross-checking*: since LLMs confabulate freely, no reference or quotation can be accepted at face value. Accordingly, every factual claim in AI content needs a human checker. At a minimum, the fact checker needs to confirm that bibliographic information provided by the AI is correct. Also, the content checker should confirm that claims made by AI are in fact supported by the referenced sources. And finally, the checker needs to confirm that any text generated by AI neither duplicates nor closely paraphrases texts from other sources. All of this is essential, and so must be explicitly confirmed, also at the head of the paper. An author can fill this role, but likewise a research assistant can contribute. In either case, that individual should be identified along with contact information in case questions arise. In this way, authors and fact-checkers are identified as explicitly and transparently responsible for the oversight of AI-generated content.
Standard 4: *intra-textual clarity*: all AI-generated content within a research report or any other publication must be set off from human-generated content through distinct style markers, like block quotations or alternate fonts.

Box 1 **Sample paper header.**

**Recent research in creativity in LLMs**

By D E Lloyd^**^, M Sharpe^*^, and P Marlowe^*^

With AI content by Open AI 4o, generated on 4_10_25 appearing in sections 2 and 3 (prompt in supplemental data)

AI content check by V I Warshawski^**^

^**^ affiliations with email addresses ^*^affiliations

These are relatively simple guidelines, readily adaptable to various contexts. Certainly research in AI and related fields should be governed by these epistemic guardrails. However, these expectations can apply to any writing presenting evidence and argumentation in support of a conclusion. They should be automatic and second nature in academic writing. Likewise, they should be taught as part of expository writing, coequal with proscriptions against plagiarism and other forms of academic dishonesty. As such, epistemic responsibility with respect to AI can become part of the school curriculum, and apply explicitly to both students and teachers.

The guidelines apply by a straightforward analogy to creative contexts as well. Artistic works may not have the production of knowledge as an immediate goal, but originality and authorship are nonetheless threatened by surreptitious AI. Artists' statements routinely accompany works of art in all mediums. These statements should explicitly meet the standards outlined above.

The guidelines here differ from those of COPE, the Committee on Publications Ethics, which broadly considers good practice in every phase of publication (COPE Council, 2023). COPE emphatically rejects co-authorship of humans and AI, and thus recommends against listing AI applications or programs on title pages. Instead, AI use should be detailed in Methods sections. However, this invites some obscurity, burying the crucial AI inflection deep in a paper. In contrast, the guidelines recommended here make AI content immediately obvious to all. As the notional example demonstrates, this frontend prominence does not imply that AI is a co-author. The recommended AI notice is distinct (and new) default information at the head of any publication.

Similarly, the International Committee of Medical Journal Editors (ICMJE) agrees with the COPE prohibition of AI co-authorship and advises writers to use AI judiciously in writing and editing (American Medical Writers Association, 2023). This also affords some latitude in acknowledging AI-generated content, leaving room for ambiguity in a reader's mind. In contrast, the guidelines here explicitly force acknowledgment of AI content and require the clear demarcation of its presence in any publication. Moreover, mandating the publication of the relevant prompts allows for easier replication and further exploration of potential AI contributions. Finally, the guidelines mandate explicit acknowledgment that AI content has been fact-checked, and elevate the role of fact-checker, thereby holding both author and fact-checker responsible for ensuring the reliability of such content.

As these guidelines become community expectations, they will help to ensure the judicious use of AI. This in turn might lessen the anxiety that sources of information are surreptitiously infected with confabulated AI content. In an ideal future, AI-generated content would be as reliable as human research. But in the real world, AI confabulation will continue to threaten our understanding of the world with its flood of accidental or deliberate fakery. Resisting this trend, the proposed benchmarks for explicit AI usage can clarify and solidify human responsibility and authority in the production of knowledge.
